# Sialendoscopy as treatment of face aesthetic surgery complications: technical note^☆^

**DOI:** 10.1016/j.bjorl.2026.101821

**Published:** 2026-04-16

**Authors:** Giulianno Molina Melo, Rafael Dias Romero, Marcello Rosano, Julia Geraldo Vieira, Murilo Catafesta Neves

**Affiliations:** a Universidade Federal de São Paulo, Escola Paulista de Medicina (UNIFESP/EPM), Department of Otorhinolaryngology and Surgery of Head and Neck, São Paulo, SP, Brazil; b Hospital Beneficência Portuguesa de São Paulo, Department of Head and Neck Surgery, São Paulo, SP, Brazil

**Keywords:** Plastic surgery procedures, Facelift, Salivary Gland Fistula, Salivary Gland Injuries, Natural Orifice Endoscopic Surgery

## Abstract

**Objective:**

The demand for cosmetic surgery on the face and body has increased worldwide. Brazil is currently the world leader in open plastic surgery and second on facelift, buccal fat removal, injectables (fillers), and thread lifts. The incidence of injuries to salivary glands, ducts, and facial nerves related to cosmetic procedures is uncommon but devastating. Treatment includes dressings, botulinum toxin, anticholinergics, drainages and salivary gland resection. Sialendoscopy has been successfully described for the treatment of ductal diseases with gland preservation. We present five cases of salivary duct injury after facial cosmetic procedures treated with sialendoscopy (gland preservation) and review of the literature.

**Method:**

Retrospective single-center case series of 5 patients who underwent sialoendoscopy, with/without combined open access, for salivary duct lesions treatment following facial aesthetic procedures. Clinical data, type of procedure, ductal lesion and outcome were examined. The diagnosis was based on clinical history, procedure, clinical and radiological examination. Our Protocol Technical Note of Sialendoscopy and Combined Access is described.

**Results:**

228 sialendoscopies were performed in 11y period, only 5 (2.2%) for complications from facial cosmetic procedures. 80% women, age 48 y. Clinical presentation: facial edema, painful bulging, secretion from incision, facial paralysis. Fillers were used in 80%, 20% underwent buccal fat pad removal and 40% deep-plane facelift. Ductal injury occurred in 100%, 40% total section, 20% partial section, 20% facial nerve injury. Sialendoscopy treatment were used in all cases, 40% in fillers and 60% associated with face lifting open access. Salivary gland preservation was achieved in 100% patients. To date, only 27 patients have been described in the literature.

**Conclusions:**

Sialendoscopy as soon as possible remains a treatment option for preserving gland function with a high success rate and minimal complications. The favorable outcome reflects the team's experience in managing face aesthetic surgery complications with the use of sialendoscopy.

## Highlights

• Sialendoscopy treats successfully facial cosmetic surgery salivary duct injury.
• There is 100% salivary gland function preservation using sialendoscopy.
• Facial cosmetic Surgery complications are rare, likely underreported globally.
• Our study adds 5 new cases of sialendoscopy; 27 cases in total reported to date.
• We strongly recommend early sialendoscopy for these cosmetic complications.

## Introduction

The demand for cosmetic surgery on the face and body has increased worldwide. In the US, there were 6.165 million procedures in 2024,^1^ with approximately 384,027 facial surgeries (including blepharoplasty, rhinoplasty, facelifts, buccal fat removal, and lip augmentation), representing a significant increase compared to 2010.^2^ Brazil is currently the world leader in open plastic surgery, with 3.12 million procedures, approximately 662,675 of which were performed on the face, where facelifts are among the most sought after (approximately 121,494 facelifts in 2024).^1^

Other cosmetic procedures such as injectables (fillers, botulinum toxin, biostimulators) are also widely performed by both physicians and non-physicians; in 2024 alone, 4.165 million were performed in the US; of these, 1.794 million were botulinum toxin, 1.229 million were hyaluronic acid, and 97,758 thousand were calcium hydroxyapatite.^1^ In Brazil, the same reality is seen with approximately 769,245 thousand injectable procedures, most botulinum toxin, followed by hyaluronic acid, and calcium hydroxyapatite, among others.^1^

Other minimally invasive procedures, such as facelifts with support threads (absorbable filaments inserted under the skin to create a lifting effect, such as PDO, PLLA, and PCL) are increasingly used in cosmetology and aesthetic medicine, being very used in Brazil.^3^, ^4^, ^5^, ^6^

There are differences and variations between open surgical techniques, as well as between techniques and types of support threads, which may explain the increased risk of complications:

- The Conventional facelifts only reposition the skin and SMAS (Superficial Musculoaponeurotic System, which tapers from lateral to medial in the middle third of the face, ending at the lateral edge of the zygomaticus major muscle) on the face;
- The Minilifts, which are smaller in scale, are recommended for mild, localized sagging; - The Temporal lift raises the lateral portion of the eyes and eyebrows;
- The Forehead lift improves forehead wrinkles;
- The Facial-neck lift serves multiple purposes on the face and neck;
- The Buccal Fat pad removal (open or transoral): removes part or all of the Bichat fat;
- The Deep plane acts deeply on the SMAS, where the surgeon detaches and repositions the deep ligaments of the face, in a line that crosses from the angle of the jaw to the side of the ocular canthus, responsible for the formation of furrows (marks) with anchoring of the tissue sagging that occurs with aging. However, this more aggressive approach to facial rejuvenation puts the parotid gland (tissue, duct, and facial nerve) at increased risk of iatrogenic injury.^4^^,^^7^, ^8^, ^9^

The incidence of complications in salivary glands and nearby (duct and facial nerve) related to cosmetic procedures (the application of fillers, open rhytidectomy procedures, buccal fat removal, deep plane facelifts, and mini-lifts with support threads) is rare in the medical literature. These lesions can be parenchymal, ductal, or in facial nerve branch in nature (0.8%–4.5%)^10^^,^^11^; for ductal lesions with salivary fistula the incidence varies between 3.1% and 6.1%^12^ and for facial nerve lesions, it varies from <1% to 4%.^11^

These complications occur due to manipulations near the parotid gland, duct, and facial nerve, especially when there is deep dissection near these structures, fixation of the subcutaneous tissue at a deeper level, resection of tissue (Bichat fat or parotid tissue), formation of granulomas or fibrosis caused by fillers with compression of the duct and nerve, and deepening of the passage of the threads to define the facial contour with penetration of the duct, nerve branches, and salivary tissue.

Treatments for these complications range from clinical observation, compression dressings, duct ligation, application of botulinum toxin, use of atropine and scopolamine, drainage punctures, open surgical drainage, reoperation to remove sutures, sialendoscopy with stent and duct reconstruction, and surgical gland resection with parotidectomy.^2^^,^^10^^,^^13^^,^^14^

Sialendoscopy was introduced in the early 1990s for the diagnosis and treatment of salivary gland diseases, initially for benign obstructive diseases, like stones and strictures, being a more conservative alternative to surgical gland resection (parotid or submandibular), with its effectiveness and use widely proven in recent years by several authors.^13^, ^14^, ^15^, ^16^, ^17^, ^18^, ^19^ The main focus is to preserve the gland and salivary function in a functional salivary gland with minimally invasive technique.

The use of sialendoscopy for the treatment of complications from cosmetic facial surgery has been successfully performed by several authors recently, an alternative to definitive salivary gland resection with parotidectomy in salivary duct lesions.^13^^,^^18^, ^19^, ^20^, ^21^

The Sialendoscopy has been used primarily as pure mode or with combined minimally invasive access in face, to allow the duct identification, stent use to salivary drainage, duct suture and surgical field drainage.^20^

In this article, we present five cases of salivary duct lesions after cosmetic procedures on the face, where sialendoscopy was used as definitive treatment, and a review of the literature.

## Methods

Retrospective single-center case series of 5 patients who underwent sialoendoscopy, with or without combined minimally invasive access, for the treatment of salivary duct lesions following facial aesthetic procedures, in a single-center, cohort study spanning 11-years (January 2014 to July 2025).

Clinical data were collected from medical records of patients who underwent sialoendoscopy for obstructive or iatrogenic diseases of the salivary glands (submandibular and parotid glands), with or without minimally invasive open procedures.

### Inclusion criteria

• All patients with salivary gland injury (parenchymal or ductal) secondary to facial aesthetic procedures in the submandibular and parotid glands who required sialendoscopy as a form of treatment.
• Complete clinical data in medical records.
• All patients who agreed to participate in the study by signing the Informed Consent Form.

### Exclusion criteria

• Loss of follow-up or follow-up of less than 2-months.
• Incomplete medical records.
• Not operated on by the same team.
• Patients who refused to participate in the study.
• Patients without indication for sialendoscopy.
• Patients in whom sialendoscopy could not be performed due to anatomical or technical changes.
• Patients previously operated on for pathologies in larger salivary glands.
• Patients who had previously undergone radiotherapy for Head and neck cancer or radiodine for PTC.

Approval from the institutional ethics committee was obtained. Data were extracted from medical records and surgical notes of patients who signed the informed consent form. The approval number from the Ethics Committee (CAAE) of the Hospital Beneficência Portuguesa de São Paulo was XXXXX, dated October 2018.

The study was conducted in accordance with the Declaration of Helsinki.^22^ This study also complies with the criteria of the Preferred Reporting of Case Series in Surgery (PROCESS),^23^ Strengthening the Reporting of Cohort Studies in Surgery (STROCSS),^24^ and the guidelines of the Standards for Quality Improvement Reporting Excellence (SQUIRE 2.0).^25^

The diagnosis of salivary duct, tissue lesions, and facial nerve branches was made through a targeted clinical history, physical examination and imaging exams such as dermatological ultrasound, magnetic resonance imaging digital sialography and computed tomography, performed in all of our patients.

To Literature Review, we perform the search on the Medline (PubMed), Embase, Cochrane Library, and Lilacs platforms, based on specific terms related to the intervention (sialendoscopy, sialoendoscopy) and specific terms related to the disease (facelift, lifting, duct injury, duct lesion, plastic surgery, filler injections, injection techniques, thread lift, thread lifting, aesthetic medicine, dermal fillers), according to the rules of the PICO search strategy^26^ and analysis by the PRISMA guideline^27^ looking to related articles.

All sialendoscopy procedures were conducted according to the literature, with the same surgical team; interventions for diagnosis and treatment have done using a sialoendoscope and supporting material, following established standards.^13^^,^^28^, ^29^, ^30^

Our objective assessment was the office based salivary gland ultrasound and the salivary flow visualization at clinical exam in the office (Table 1).

**Table 1:** Clinical characteristics of patients with iatrogenic facial injuries after cosmetic procedures and clinical outcome.

Case Feature	1	2	3	4	5
Age	37	80	46	43	38
Gender	M	F	F	F	F
Cosmetic Procedure	Buccal Fat Pad Resection	Facial Fillers (Silicon)	Facial Fillers (PMMA)	Deep Plane Lifting + Fillers (hyaluronic acid)	Deep Plane Lifting + Fillers (hyaluronic acid)
Time from Procedure	14 days	12 months	10 months	15 days	45 days
Comorbidities	None	Systemic Arterial Hypertension, Diabetes Mellitus	None	None	None
Sialendoscopy Date	03/12/22	15/7/23	13/11/24	02/07/25	17/07/25
Location injury	Parotid Duct	Parotid Duct	Parotid Duct	Parotid Duct, Salivar tissue, Buccal branch facial nerve	Parotid Duct
Side	Rigth	Left	Rigth	Rigth	Left
Symptoms	Swelling/pain/fistula/ skin hyperemia	Swelling/pain/ skin hyperemia	Swelling/pain/xerostomia/ skin hyperemia	Swelling/pain/fistula/ skin hyperemia, facial paralisis	Swelling/ Pain/ Fistula/ Skin hyperemia
Diagnostic Exam	SialoRM	SialoRM/ Dermatologic Ultrasound	SialoRM/ Dermatologic Ultrasound	SialoRM	SialoRM
Lesion Type (Nahlieli^20^	Type 4 (Total)	Type 3 (Striture)	Type 3 (Striture)	Type 4 (Total)	Type 4 (Partial)
Treatment	Sialendoscopy with Stent and drainage with marsupialization for oral cavity by Combined Procedure	Sialendoscopy, dilations, stents, intraductal corticosteroids	Sialendoscopy, dilations, stents, intraductal corticosteroids	Sialendoscopy, Neoduct reconstruction using stents by Combined Procedure	Sialendoscopy, Neoduct reconstruction using stents by combined procedure
Outcome	Asymptomatic, Gland preserved, Partial stenosis of papila, with saliva drainage through ostium	Asymptomatic, Gland preserved, Complete remission of symptoms	Asymptomatic, Gland preserved, Complete remission of symptoms	Asymptomatic, Gland preserved, Complete remission of salivary symptoms Facial nerve paralysis (buccal branch)	Asymptomatic, gland preserved, complete remission of symptoms
Time to Resolution	4 months	2 months	4 months	2 months	2 months
Objective Assessment Result	Office Ultrasound: normal gland Physical exam: less salivary flow on manual expression	Office Ultrasound: normal gland Physical exam: normal salivary flow on manual expression	Office Ultrasound: normal gland Physical exam: normal salivary flow on manual expression	Office Ultrasound: normal gland Physical exam: normal salivary flow on manual expression, Facial nerve paralysis (buccal branch)	Office Ultrasound: normal gland Physical exam: normal salivary flow on manual expression
Follow up (time months)	35	27	11	4	4

Oded Nahlieli, et al. Endoscopic Treatment of Salivary Gland Injuries due to Facial Rejuvenation Procedures.2008,118(5),763−767. doi:10.1097/mLg.0b013e31816381.^20^

### Sialendoscopy technique

Our Protocol was previously described^29^ and the main steps are:

The patient in locate in the supine position under general anesthesia with the orotracheal tube located on the contralateral side of the affected gland for sialendoscopy. Routine mouth asepsis and antiseptic following the rules with proper oral 0.2% chlorhexidine mouthwash and covering the sterile surgical field, ensuring that the side on face and oral cavity which sialendoscopy to be performed is exposed.

**Fig. 1:**
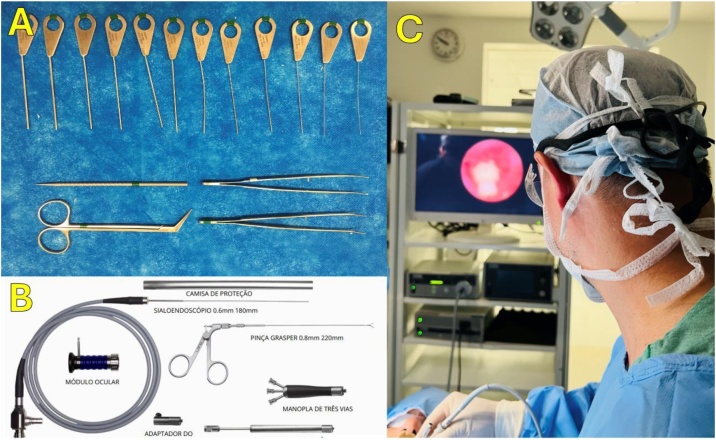
Sialendoscopy materials. (A) Support material: dilators, forceps, scissors. (B) Sialendoscope, forceps, handle, video adapters, fiber optic adapter. (C) Video rack, Video monitor, Light source, Video camera, Sterile fields.

All instruments necessary to the sialendoscopy procedure is prepared: video rack, dilatators, instruments, sialendoscopes (Fig. 1).

The sialendoscope setup is checked as soon as the sterile field is ready on the patient, making the necessary adjustments: the white balance, the zoom and focus on the camera. The spatial orientation of the optical instrument, which is necessary for safe intraductal navigation is checked.

With open-mouth gag on the contralateral side, adequately exposing the papilla of the salivary gland to be submitted to sialendoscopy is made, either submandibular or parotid gland.

We perform a progressive dilatation of the salivary gland papilla with dilatators until it fits the adequate size of the sialendoscope to allow the insertion of the sialendoscope to proper visualization of the duct.

After dilatation, we introduce delicately the sialendoscope inside the main duct and connect a 20 mL syringe filled with sterile 0.9% physiological saline solution or sterile isotonic water to the irrigation system connected to the sialendoscope irrigation system. Slowly and carefully, we inject small portions, with low pressure, of sufficient volume of the sterile 0.9% physiological saline solution to distend the duct that will be visualized on the video monitor screen.

Once this step is made, we navigate gently into the duct once it is distended, allowing the sialendoscope to enter it effortlessly without major maneuvers and twists. Now we inspect the main duct, secondary and tertiary ducts as possible, in detail with sialendoscope, cleaning from mucus plugs using the saline solution irrigation instilled by the syringe, trying to locate the duct stricture, laceration or complete duct cut.

### Sialendoscopy and combined access: protocol technical note

**Fig. 2:**
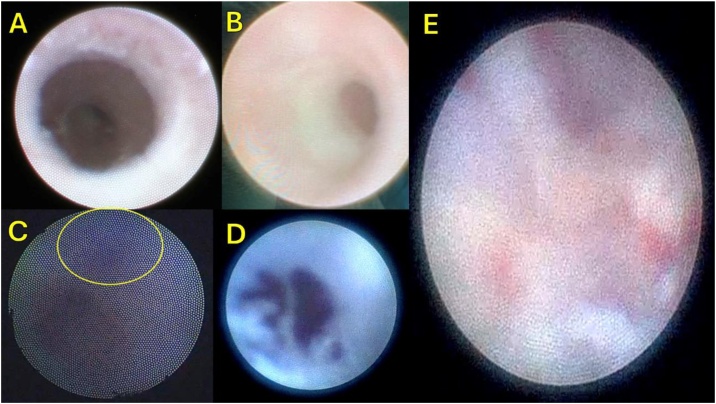
Appearance of the duct by sialendoscopy view in iatrogenic lesions caused by cosmetic procedures. (A) Normal Duct Appearance in Sialendoscopy. (B) Stenosis due to extrinsic compression by silicone. (C) Partial Duct Lesion in Sialendoscope view showing the 4‒0 violet poliglactin suture transfixed (yellow circle) after Deep plane surgery. (D) Sialendoscopic view of total duct section after deep plane Surgery (E) Appearance of the extraductal soft tissue area visualized by the sialendoscope after passing through the total duct section.

Once we confirmed the duct lesion by external compression (fillers), we initially perform the pure sialendoscopy to inspect the duct, clean mucous and debris, insert a guide wire through the endoscope to do mechanical dilation, followed by a balloon in the stenotic portion to be inflated and perform mechanical dilation (for approximately one minute). Then, a 3 mL of steroid (hydrocortisone) are instilled over another one minute, and finally, a stent of appropriate size is placed inside the duct, extending from proximal part in the parotid gland through the papilla into the oral cavity, whose will be removed after 45-days. The main objective is to open the ductal stricture and derivate the saliva to drainage it into the mouth. This was done in all fillers complication cases (Fig. 2B).

**Fig. 3:**
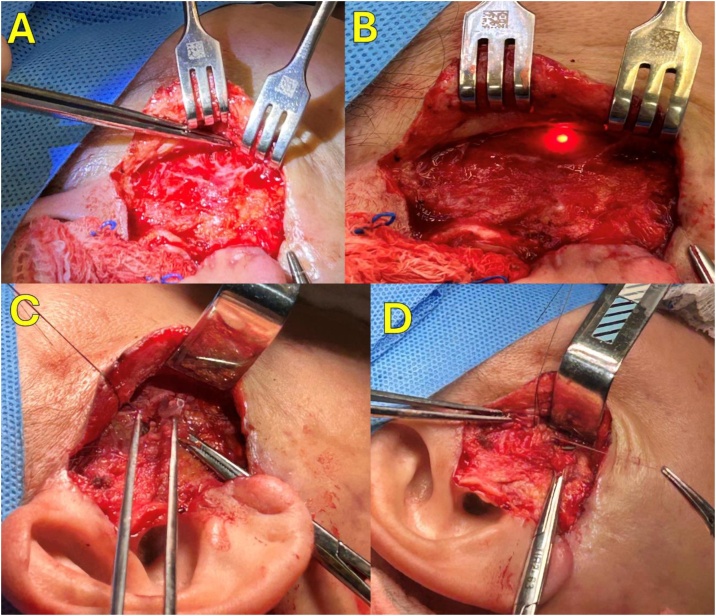
Duct reconstruction using a sialendoscope and combined facial access. (A) Combined Facial Access to locate the Sialendoscope in the total duct section area. (B) Combined Facial Access with Sialendoscope viewed in the total duct section area (brightness of light). (C) Passage of the salivary stent through the duct with the aid of the sialendoscope and visualized in the Combined Access area (between the forceps). (D) Reconstruction of Neoduct around the salivary stent.

In cases of duct lesion (transfixed lesion or total/partial cut) confirmed intraductally by the sialendoscope (Fig. 2C and D), we perform de minimally combined access through the same facial incision of rhytidectomy (lifting), open the skin flaps exposing the parotid area, guided by the sialendoscopy light and by palpation of the endoscope to locate and expose the main duct and the duct lesion region (Fig. 3A and B).

The lesion is confirmed again by direct visualization of the sialendoscope ligth in the operative filed, by visualization of the sialendoscope itself or by the visualization of the instilled fluids through the endoscope in the duct perfuration (Fig. 3B).

**Fig. 4:**
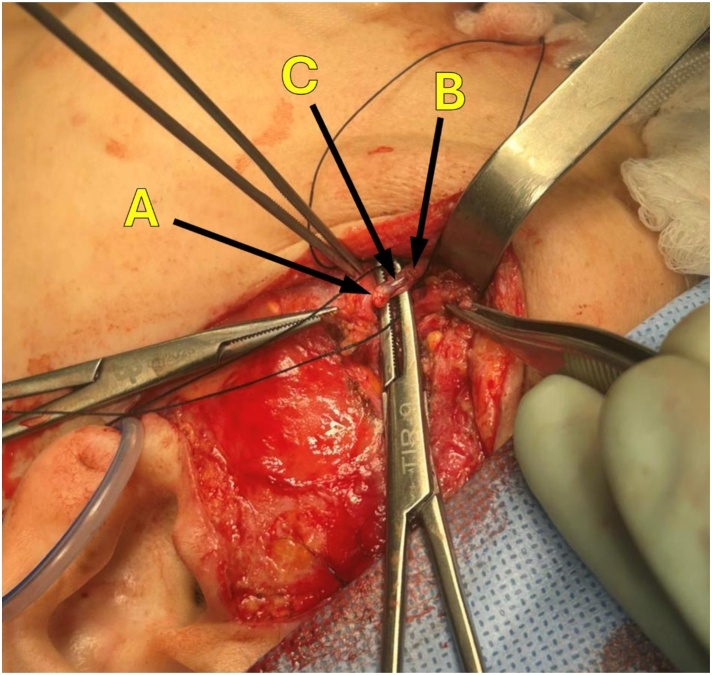
Total duct lesion reconstruction using sialendoscope, silastic stent and combined facial access. (A) Proximal parotid duct stump. (B) Distal parotid duct stump, salivary stent located intraductally. (C) Silastic Stent located intraductally.
3) In case of approximation of the stumps are not possible due high tension in the suture, we reconstructed the duct creating a neo duct around the salivary stent who communicated both proximal and distal duct stumps, using local tissues (parotid and fat nearby), with nylon 6‒0 or polyglactin 5‒0 sutures with surgical loupes and local drainage was done with passive drain (Fig. 3C and D).

Then, once the partial duct lesion is noted or the proximal and distal stumps are identified in total cut lesion, they are dilated with the endoscope and the reconstruction of duct begin:

1) In case of partial lesion of the duct, if there is previous suture, it was removed and a duct reconstruction with new suture was done with nylon 6‒0 or polyglactin 5‒0 sutures using surgical loupes, with sialendoscope inside the duct, who were inspected with the sialendoscope to cleanning from plugs, locate the stent and at the end, local drainage was done with passive drain (Fig. 2C).
2) In case of total cut lesion, if there is easily approximation of stumps without tension, we reconstructed the duct with salivary stent inside located with sialendoscope help, sutured the duct in a circle way with nylon 6‒0 or polyglactin 5‒0 sutures using surgical loupes, then local drainage with passive drain are imperative (Fig. 4).

**Fig. 5:**
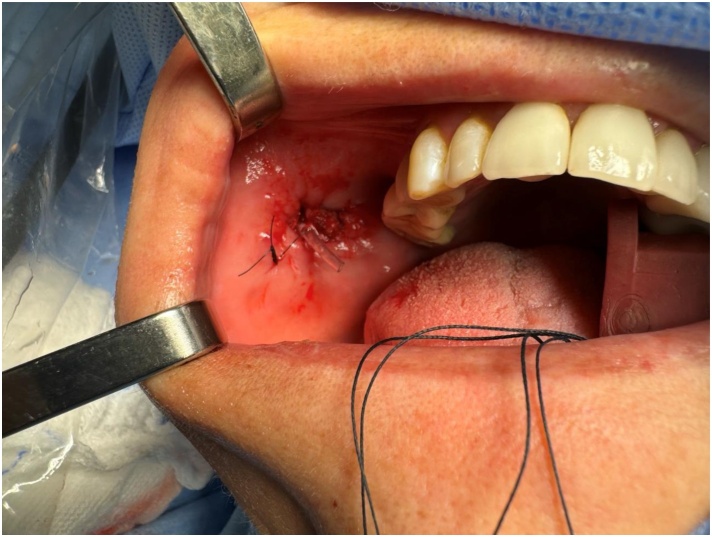
Appearance of the end of the salivary stent fixed to the oral mucosa.

In all cases the distal end of stent was sutured to the buccal mucosa with nylon 4‒0 (Fig. 5).

Our technical protocol include the intraoperative, intraparotid botulinum toxin injection to allow a good healing of the ductal sutures, decreasing the salivary fistula risk. We used a 100 U dilluted to 3 mL of saline solution, distributed in 6-points inside the gland (3-points in upper pole and 3-points in the inferior pole).

In the post-operative period, all patients have the salivary stent placed for 45-days and the passive drain for 10-days. Our preferred salivary stent is the pediatric urethral probe catheter, number 4Fr or 6Fr, made from silastic which has good resistance and malleability (Fig. 4).

Our Post Sialendoscopy and Duct Reconstruction Protocol is to maintain endovenous antibiotics for two days (cefalosporines 2.generation), soft diet on the next operative day (without citrics), in all cases. Also, we have prescribed hioscin 1 tablet 3 times a day as anti-sialogogue, during 2- to 4-weeks.

All patients were discharged on second postoperative day, without other interferences to regular biweekly ambulatory follow-up in the first moth and then monthly until 6 months for clinical oral examination.

On second month, we perform an office based ultrasound and clinical oral examination to check the salivary flow inside the duct.

Our objective assessments were the time to resolution, presence of salivary flow on clinical oral examination after two months from the procedure and the salivary gland ultrasound in the office.

## Results

During the 11-years period, 228 sialendoscopies were performed for obstructive or iatrogenic salivary gland diseases, of which 35 were for stenosis, 189 for sialolithiasis, and only 5 (2.2%) for the treatment of complications from cosmetic procedures on the face.

The clinical characteristics, cosmetic procedure performed (fillers, surgical, threads), diagnostic tests, treatment, type of lesion, follow-up time, and clinical outcome of these patients are described in Table 1.

There were 4 women, mean age of 48y (37‒80 y). The clinical presentation included edema and painful bulging in the parotid region, serous secretion from the incision, skin hyperemia, local pain and in one case, facial paralysis.

**Fig. 6:**
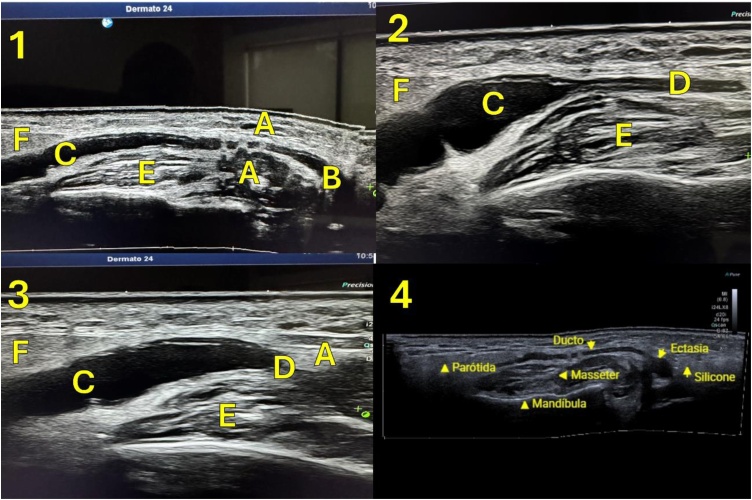
Dermatological ultrasound with stenosis and ectasia of the salivary duct due to compression by filler (silicone). (A) Silicone filler. (B) Duct. (C) Duct ectasia. (D) Duct stenosis. (E) Masseter. (F) Parotid.

**Fig. 7:**
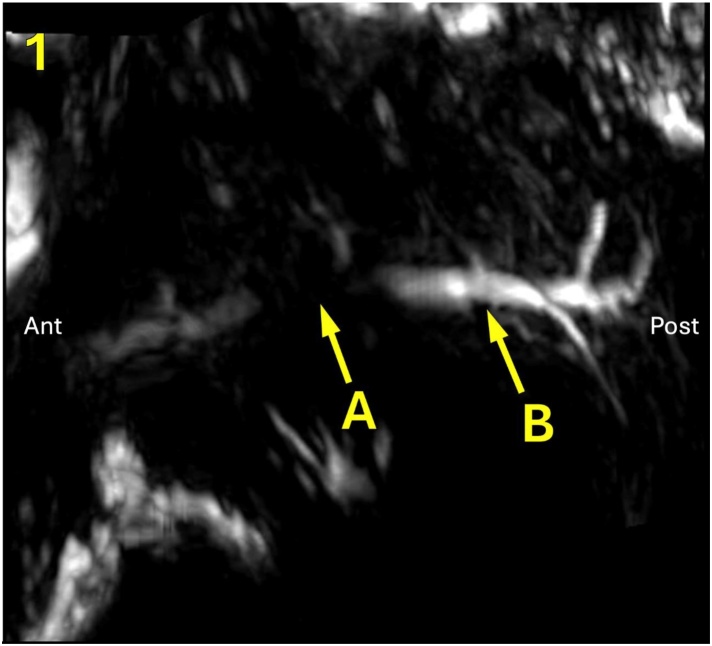
Magnetic resonance imaging showing fillers causing parotid duct stenosis: MRI profile, sagittal section with parotid duct stenosis due to compression by filler (Silicone). (A) Duct filling failure. (B) Proximal duct dilation.

**Fig. 8:**
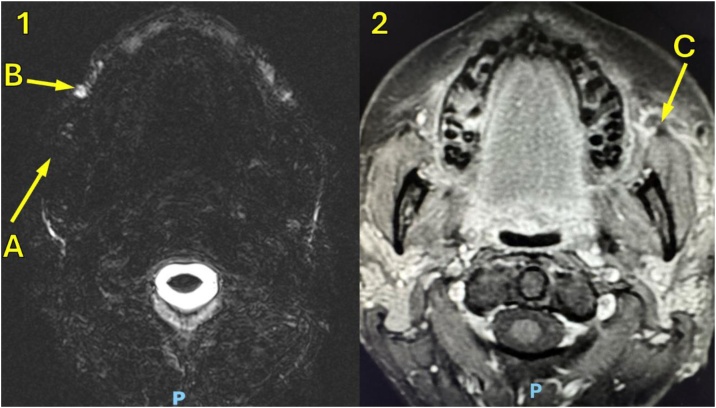
(1) Axial MRI, T2, with filling material (Silicone) occluding the parotid duct by compression. (A) Duct filling failure. (B) Filling material (Silicone). (2) Axial MRI, T1with partial parotid duct lesion in the anterior border of masseter muscle and salivary fistula. (C) Partial Parotid Duct lesion with salivary fistula.

The diagnosis of salivary duct, tissue lesions, and facial nerve branches was made through a targeted clinical history, physical examination, and imaging exams such as dermatological ultrasound (Fig. 6), magnetic resonance imaging (Fig. 7, Fig. 8**)** and computed tomography, performed in our patients whose showed strictures or ductal lesions.

The fillers were used by 80% (4/5) of patients (hyaluronic acid, silicone and PMMA), all were office based injected without guided ultrasound, at different depths, by physicians and non-physicians. One patient underwent office based buccal fat pad removal by non-physicians and two underwent a deep-plane facelift in operative room under general anesthesia by Plastic Surgeons.

There was ductal injury in all cases, 2 patients with total section, one associated with parotid injury, and one with facial nerve (buccal branch) injury.

The most severe injuries: Nahlieli type 4, occurred in patients undergoing buccal fat removal and deep plane facelifts, and Nahlieli type 3 injuries occurred in patients undergoing filler procedures^20^ (Table 1).

Sialendoscopy was used as treatment in all cases, 2 with Fillers cases and 3 associated with face lifting open access, all with salivary gland preservation (100% of success). The time to resolution varied from 2-months to 4-months (Table 1).

The Fig. 2 shows our sialendoscopic view findings in salivary duct injuries in cosmetic procedures. One have to note the intraductal stricture due external compression (inflamatory) due fillers (B), transfixant suture (violet poliglactinin) in partial duct lesion (C), total duct section after deep plane Surgery (D) and the appearance of the extraductal soft tissue area visualized by the sialendoscope after passing through the total duct section (E).

The Fig. 3 demonstrates how the open access (facial lifting) procedure was performed in combination with sialendoscopy to the reconstruction of the salivary duct with stent use, as described in the Protocol Technical Note above.

The Fig. 4 shows the total duct lesion reconstruction using sialendoscope, stent and combined facial access.

And the Fig. 5 shows the final appearance of the duct with the stent fixed in the oral cavity at the end of the surgery.

Our Literature search has resulted in a compiled a list of cases with updates from the medical literature in which sialendoscopy were used as main treatment in aesthetic face procedures complications, including the five cases in this article, totaling only twenty-seven cases to date.

We have found normal salivary gland appearence on the ultrasound in all patients at the time of resolution, with good salivary flow visualization at clinical exam in 100% of patients (Table 1).

## Discussion

With the increasing demand for cosmetic surgery on the face and body worldwide, including Brazil, which is the world leader in open plastic surgery, the occurrence of iatrogenic complications tends to increase dramatically. To present, few complications are reported in the literature, probably many of them are not published and the real number of cases may be much higher.

Facelifts (and their variants), support threads, buccal fat removal, fillers, and deep plane techniques present an increased risk of injury to the salivary glands, ducts, and facial nerve.^1^^,^^2^^,^^10^

Common facelifts and facial procedures complications are sialoceles, salivary fistula, salivary duct injury, facial nerve injury, hematomas, prolonged edema, obstructive parotitis, and xerostomia.^10^^,^^11^^,^^20^^,^^31^, ^32^, ^33^, ^34^

Some data reports in the medical literature on face complications are related to parotidectomies performed for oncological indications or benign lesions, and not directly to cosmetic facelift techniques.^5^^,^^6^^,^^10^^,^^32^

However, the deep-plane and facelift technique involves dissection and manipulation near the parotid tissue and surrounding areas, as well as the passage of support threads, so the risk to injuries on the parotid tissue, parotid duct and branches of the facial nerve, is real and may increase alarmingly in certain situations: inexperience of the surgeon, lack of knowledge by non-physicians, anatomical variations, alteration of the site due to previous inflammatory processes (fillers, previous manipulation), attempt at more aggressive rejuvenation.^8^^,^^12^^,^^33^, ^34^, ^35^, ^36^, ^37^, ^38^, ^39^, ^40^

Bichectomy (buccal fat pad removal), a surgery commonly performed in Brazil, presents a high risk of complications when performed by inexperienced hands and by non-medical professionals. Complications arise from a false perception of safety and a lack of knowledge of anatomy,^9^ as these procedures are often performed by untrained and non-medical personnel in non-surgical environments. Treatment varies according to the injury, with sialocele and salivary fistula occurring mainly due to injury to the salivary duct and, not infrequently, injury to the buccinator (buccal) branch of the facial nerve.^41^, ^42^, ^43^, ^44^ The diagnosis is made based on the clinical history, clinical examination, imaging tests, and amylase dosage in the puncture fluid.^43^ Treatment varies from clinical with compression dressings, botulinum toxin application, sialendoscopy, and surgical reapproach.^44^ In our case series, one patient presented with duct injury due to buccal fat removal, with total duct sectioning, and was successfully treated with combined sialendoscopy, duct reconstruction, and drainage.

Complications involving facial fillers (mainly hyaluronic acid) in the parotid gland and branches of the facial nerve are rare, but serious, and are more related to the injection technique in risk areas and to local fibrosis and necrosis that may involve surround structures (branches of the facial nerve, parotid duct, parotid salivary tissue, and arterial supply).^45^^,^^46^ Deep and inadvertent injection into regions adjacent to Stensen's duct (cheek region) can cause direct injury or compression of the salivary duct and facial nerve, resulting in obstructive sialadenitis of the parotid gland, facial paresis and even tissue necrosis.^47^^,^^48^ Late injuries, such as those in the patients who underwent filling in this article, are related to a history of local chronic inflammation that favor the formation of granulomas and biofilms, putting pressure on nerve or ductal structures.^49^^,^^50^

The diagnosis of aesthetic procedures complications in salivary duct, tissue lesions, and facial nerve branches is made through a targeted clinical history, physical examination, and imaging exams such as dermatological ultrasound, magnetic resonance imaging and computed tomography, performed in our patients, as shown in Table 1, Fig. 6, Fig. 7, Fig. 8.

In our cases, the clinical presentation included edema, painful bulging in the parotid region, skin hyperemia, and in one case, facial paralysis, as already described in medical citations.^51^, ^52^, ^53^, ^54^, ^55^

There are different treatments in the literature to these complications: open reconstruction, botulinum toxin-based conservative management, duct ligation, conservative measures alone and salivary gland resection. The utility of sialendoscopy in the treatment of aesthetic surgery complications is based mainly on gland preservation: as auxiliary technique in open duct reconstruction and as main technique in intraductal compression or strictures, however, fewer reports still exist on literature, probably due to the reduced number of sialoendoscopy specialists who act as reference centers worldwide (Table 2).

**Table 2:** Literature review of cases of iatrogenic salivary duct injury following cosmetic procedures treated with sialendoscopy.

Number	Author	Year	Patient (N)	Type of Injury^20^	Treatment (Pure Siale, Combined Siale) (N)	Outcome (N) of preserved glandular function Yes/No	Reference
1	Nahlieli et al.	2008	14	2‒4	Pure Siale	11 Yes	[20]
					8 dilatations	3 No	
					4 stents		
					2 compressive dressing		
2	Melo et al.	2025	5	3‒4	Pure Siale: 2 dilatations & stent	5 Yes	Present article
					Combined Siale: 3 reconstruction & stent		
3	Zheng et al.	2025	3	4	Pure Siale: 3	3 Yes	[38]
4	Choi et al.	2019	2	3	1 Pure Siale: dilatations	2 Yes	[34]
					1 Combined Siale		
5	Iwai et al.	2020	1	4	Pure Siale: 1	1 Yes	[64]
6	Barron et al.	2001	1	2	Combined Siale: 1	1 Yes	[65]
7	Kopec et al.	2013	1	3	Combined Siale: 1	1 Yes	[63]

Sialendoscopy was used for the diagnosis and treatment of salivary gland diseases, initially for obstructive stones, then to duct stenosis, being a conservative alternative to definitive surgery to remove the glands. Its effectiveness and widely use has been proven in recent years by several authors, with very satisfactory results.^13^, ^14^, ^15^^,^^17^^,^^28^^,^^56^

The sialendoscopy technique consists of introducing a sialendoscope, coupled with optics and a working channel, with a diameter ranging from 1.3 mm to 1.6 mm, through the ostia of the salivary papillae to inspect the ducts and treat pathologies of the salivary ducts^13^^,^^15^ (Fig. 1).

Other indications have appeared with great certainty, based on large experienced use of the sialendoscopy technique, such as treatment of aesthetic surgery iatrogenic salivary duct lesions, as those presented in this article, with excellent clinical results given the preservation of the gland and evident improvement in the patient's quality of life.^29^^,^^57^, ^58^, ^59^, ^60^

However, there is a lack in the literature on this topic, with few studies presented (Table 2), mainly due controversy that are few reported aesthetic complications (is there really few complications or are these not reported?) and to the treament with other conservative techniques or non-conservative open procedures by non sialendoscopy experts, indeed, our experience can add some helpful tips to the sialendoscopy practioner and to the medical literature aiming to preserve the gland.

The usual findings in sialendoscopy for obstructive sialadenitis are: mucus plugs, pale ductal mucosa, stenosis, various types of stones, duct fibrosis and narrowing of ducts; but, in iatrogenic lesions, ordinarily we can find stenosis due to extrinsic fillers compression, stenosis due suture duct transfixation, partial duct lesion, and total duct section^14^^,^^57^ (Fig. 2).

The treatment of ductal injuries can be performed using sialendoscopy alone or in combination with minimally invasive extraoral access (face lifting) to assist and complement ductal treatment by opening the skin to safely reconstruct the salivary duct restoring salivary physiology, used sucessfully in three cases in our casuistic (Table 1).

Sialendoscopy treats lesions by washing the ducts to clear blockages, removing mucus plugs and debris, removing foreign bodies (support wires), dilating stenotic segments, using stents in ductal stenosis, marsupialization of the duct and stent, placing stents to guide saliva flow to the oral cavity and preventing duct collapse, reducing salivary fistula (saliva leakage into tissues outside the duct) in partial lesions, using stents around tissues to create a neo-duct in total lesions, thus preserving the gland, successfully applied in the present reported cases, with satisfactory outcome (gland preservation^20^^,^^21^^,^^60^, ^61^, ^62^ (Fig. 4).

The sialendoscopy has major advantage among other alternative approaches and conservative methods in a way that almost the salivary gland can be preserved with minimally invasive technique, unlike when compared to duct ligation; and it can be used as auxiliary procedure in the open duct reconstruction, like ours cases. The botulinum toxin-based conservative management and others conservative measures alone have the disavantage that not solve the duct lesion problem, just postpone it, with risk evolution to duct stenosis due fibrosis.

In this article, all cases were treated with sialendoscopy alone or in combination with minimally invasive access, resulting in gland preservation in all of patients. Duct reconstruction was performed in 3 patients through suturing and the use of intraductal salivary stents for saliva drainage, in addition to the application of botulinum toxin to reduce salivary secretion while fibrosis (periductal or peri-stent) occurs, sealing the site (Table 1, Fig. 3, Fig. 4). In the other 2 patients, the use of stents with duct dilation allowed saliva drainage through the stenotic area from fillers compression and gland preservation, similar to other authors in the literature.^63^, ^64^, ^65^

Unlike some authors, as expert-opinion-based, we state that early intervention may be considered in the treatment algorithm, aiming fast tissues healing, without previous manipulations with fibrosis, without actual infections and with objective to prevent future stenosis in the ducts.^43^^,^^45^ Indeed, like Koch et al.^66^^,^^67^ Lewkowicz et al.,^68^ Van Sickels et al.^69^ and others, we recommend, as soon as possible, the intervention with sialendoscopy, combined or not with open access, utilizing our technical protocol and using intraoperative botulinum toxin at the end of procedure in all interventions for complications of cosmetic procedures to prevent external salivary fistula, scarring and duct strictures, with excellent outcome.^68^^,^^70^

Patients who underwent sialendoscopy for any other indications experienced an improvement in quality of life after the procedure, as demonstrated by several authors, with 85% satisfaction in the quality of life questionnaire and with a high treatment success rate.^63^^,^^71^^,^^72^ But, unfortunately, in our cases no validated functional or quality-of-life assessment tools were employed, a clearly limitation of the article; nonetheless, this will be corrected in future cases.

There are few reports in the literature mentioning the use of sialendoscopy for the treatment of iatrogenic lesions resulting from cosmetic procedures on the face. Complications often occur but are not published. Our updates compilation has resulted only twenty-seven cases to date including ours from the medical literature in which sialendoscopy were used as treatment (Table 2).

The authors emphasize that sialendoscopy has a high success rate in the treatment of ductal complications in cosmetic procedures on the face and should be indicated as soon as the lesion is clinically confirmed, with excellent benefits for the patient. The cases described here contribute to the scientific literature, so that treatment orientations can be created in the future.

This article, the first of its kind in a Portuguese-speaking country, aimed to evaluate the results of the use of sialendoscopy, combined or not with minimally invasive combined access, for the treatment of iatrogenic salivary duct lesions after cosmetic procedures on the face, proving its high efficacy and resoluteness, with preservation of the functioning gland in all patients.

## Conclusions

The results of our study demonstrate that a thorough anatomical knowledge combined with experience in facial aesthetic procedures can prevent complications involving the salivary ducts, facial nerve, and parotid salivary tissue. We recommend Sialendoscopy, as soon as possible, as option for preserving gland function, with a high success rate and minimal complications. The favorable outcome of the presented cases reflects the team's experience in managing complications with the use of sialendoscopy in face aesthetic surgery complications.

## ORCID ID

Giulianno Molina Melo: 0000-0001-8220-6317

Rafael Dias Romero: 0000-0003-3028-123X

Marcello Rosano: 0000-0002-3026-466X

Julia Geraldo Vieira: 0009-0006-2142-6838

Murilo Catafesta Neves: 0000-0002-8094-6298

## Funding

The authors declare no financial support to this article

## Data availability statement

The authors declare that all data are available in repository.

## Declaration of competing interest

The authors declare no conflicts of interest.
